# Drugs Associated with More Suicidal Ideations Are also Associated with More Suicide Attempts

**DOI:** 10.1371/journal.pone.0007312

**Published:** 2009-10-02

**Authors:** Henry T. Robertson, David B. Allison

**Affiliations:** 1 Department of Biostatistics, University of Alabama at Birmingham, Ryals School of Public Health, Birmingham, Alabama, United States of America; 2 Clinical Nutrition Research Center, University of Alabama at Birmingham, Birmingham, Alabama, United States of America; University of British Columbia, Canada

## Abstract

**Context:**

In randomized controlled trials (RCTs), some drugs, including CB1 antagonists for obesity treatment, have been shown to cause increased suicidal ideation. A key question is whether drugs that increase or are associated with increased suicidal ideations are also associated with suicidal behavior, or whether drug–induced suicidal ideations are unlinked epiphenomena that do not presage the more troubling and potentially irrevocable outcome of suicidal behavior. This is difficult to determine in RCTs because of the rarity of suicidal attempts and completions.

**Objective:**

To determine whether drugs associated with more suicidal ideations are also associated with more suicide attempts in large spontaneous adverse event (AE) report databases.

**Methodology:**

Generalized linear models with negative binomial distribution were fitted to Food and Drug Administration (FDA) Adverse Event (AE) Reporting System (AERS) data from 2004 to 2008. A total of 1,404,470 AEs from 832 drugs were analyzed as a function of reports of suicidal ideations; other non-suicidal adverse reactions; drug class; proportion of reports from males; and average age of subject for which AE was filed. Drug was treated as the unit of analysis, thus the statistical models effectively had 832 observations.

**Main Outcome Measures:**

Reported suicide attempts and completed suicides per drug.

**Results:**

832 drugs, ranging from abacavir to zopiclone, were evaluated. The 832 drugs, as primary suspect drugs in a given adverse event, accounted for over 99.9% of recorded AERS. Suicidal ideations had a significant positive association with suicide attempts (p<.0001) and had an approximately 131-fold stronger magnitude of association than non-suicidal AERs, after adjusting for drug class, gender, and age.

**Conclusions:**

In AE reports, drugs that are associated with increased suicidal ideations are also associated with increased suicidal attempts or completions. This association suggests that drug-induced suicidal ideations observed in RCTs plausibly represent harbingers that presage the more serious suicide attempts and completions and should be a cause for concern.

## Introduction

A “higher rate of ‘suicidal ideation’ has not clearly been shown to translate into a higher rate of suicide.” So wrote Deprés and colleagues when responding to concerns about suicidal ideation for the obesity drug rimonabant [1].

Suicidal ideation among patients taking certain drugs, including antiobesity drugs such as the CB1 antagonists rimonabant and taranabant and the dopamine antagonist ecopipam, have received much attention in recent years [2]–[5]. Suicidal behavior presents itself in a variety of forms, ranging from suicidal ideations to attempts and completions.

The predictive significance of drug-induced suicidal ideations remains open to question. In fact, an epidemiologic investigation of over 5,000 persons followed for 10 years found that, “Prior ideation is negatively related, though, to plan ([odds ratio; OR  = ] 0.4) and attempt ([OR  = ] 0.2) at follow-up” [6]. Thus, it is not certain that drugs which put patients at increased risk of suicidal ideations also put them at increased risk of suicidal attempts or completions. In the context of drug development, if a drug undergoing testing yields reports of suicidal ideations, but no suicide attempts, should such a drug be taken as presenting a serious suicide risk to the population at large? This is difficult to discern because even in relatively large RCTs (e.g., a few thousand people), it will be very difficult to detect effects on events as rare as suicides.

The purpose of this study was to use the non-randomized, but far more numerous AE reports maintained by the FDA to determine what associations exist, if any, between drugs associated with suicidal ideations and drugs associated with suicide attempts. The FDA's Adverse Event Reporting System (AERS) has been collecting voluntarily reported AEs since 1969, and does not provide the “denominator data” on the prevalence of drug use in the population. Nevertheless, one can still employ case-only methodologies to compare the incidence of differing reactions by drug [7]. We believe that non-suicidal adverse events serve as a suitable proxy for denominator data, and they are included as predictors in the model. AERS provides details of the drugs taken, the reactions, and demographic information. By adjusting for the total volume of AERs and demographic variables, it is possible to determine whether there exists a statistical association between suicidal ideation and attempts.

## Methods

### Data

We evaluated AERS data from 2004 to 2008, which were freely available for download from the FDA's web site [8]. We identified 832 drugs that accounted for 1,404,470 AEs, which covered more than 99.9% of all AEs that were not recreational or herbal drugs reported during the time period.

### Statistical Methods

The downloaded raw data included files on the drugs taken, demographic information about the patient, and the reaction. The files were cross-matched through unique report identifiers in the “ISR” field. The “primary suspect” drugs listed in the drug files were, depending on the report, listed by their trade names or generic names; trade names were all converted to generic names for consistency. Nutritional supplements, recreational drugs, and herbal drugs were excluded from the analysis. We thus found 832 drugs that accounted for 1,404,470 AERs, an average of 1,688 AERs per drug. The drugs were classified into one or more indications, as shown in Tables 1 and 2. While the exact classification of the drugs is in some cases debatable, drug class served as merely an adjustment in the regression models and was not the primary focus of this paper. “Antibiotics” were broadly defined to include antiviral drugs; similarly, “analgesics” included anesthetics and painkillers.

**Table 1:** Drug Characteristics.

Drug Category*	Number of Drugs	Suicide Attempts	Suicide Ideations	Suicidal Behavior	Non-Suicidal AERs	% Male	Mean Patient Age
AIDS	21	53	44	0	20053	68.6	41.0
Allergy	46	616	448	27	62,395	44.2	47.5
Analgesic	54	4,184	916	22	178,970	43.3	50.2
Antibiotic	133	642	849	14	122,690	48.2	49.2
Anti-Depressant	32	3572	2631	31	77067	40.0	47.9
Anti-inflammatory	58	501	326	10	263,450	43.4	52.9
Anti-psychotic	21	1,426	712	23	63,406	49.9	44.2
Anxiety	29	2256	757	15	33553	37.8	48.8
Cancer	99	130	134	1	167,418	50.8	55.4
Diabetes	21	204	36	0	76,459	50.7	60.5
Heart	110	934	187	7	115,242	53.2	62.8
Muscle Relaxant	45	1,773	681	12	57,989	42.4	46.2
Obesity	7	30	32	5	17079	34.5	39.6
Sleep	14	558	96	5	19,340	44.0	51.1
**Total**	**832**	**14,351**	**7,891**	**193**	**1,382,035**	**46.4**	**52.5**

* A drug can belong in more than one category. The “total” line at the bottom is not the sum of the rows above it.

**Table 2:** Drugs by category.

Category	Number of Members	Members
AIDS	21	abacavir, amprenavir, atazanavir, darunavir, didanosine, efavirenz, emtricitabine, enfuvirtide, fosamprenavir, indinavir, lamivudine, lopinavir, nelfinavir, nevirapine, ritonavir, saquinavir, stavudine, tenofovir, tipranavir, zalcitabine, zidovudine
Allergy	46	albuterol, aminophylline, azelastine, beclomethasone, budesonide, cetirizine, chlorpheniramine, cimetidine, cromolyn, cyclizine, cyproheptadine, desloratadine, dexchlorpheniramine, diphenhydramine, doxepin, doxylamine, ephedrine, epinastine, epinephrine, famotidine, fexofenadine, fluticasone, formoterol, glatiramer, hydroxyzine, ipratropium, ketotifen, levalbuterol, loratadine, meclizine, mianserin, montelukast, nicardipine, nitric oxide, nitroglycerin, olopatadine, omalizumab, phenyltoloxamine, pirbuterol, promethazine, salmeterol, terbutaline, terfenadine, theophylline, tiotropium, zafirlukast
Analgesic	54	acetaminophen, acetylsalicylate, acetylsalicylic acid, balsalazide, buprenorphine, butalbital, butorphanol, celecoxib, codeine, cyanocobalamin, diclofenac, dihydrocodeine, dipyrone, dronabinol, etoricoxib, fentanyl, gabapentin, hydrocodone, hydromorphone, ibuprofen, indomethacin, ketoprofen, ketorolac, mefenamic acid, meloxicam, meperidine, mesalamine, methadone, morphine, nabumetone, nalbuphine, naproxen, nimesulide, nitrous oxide, opium, oxaprozin, oxycodone, oxymorphone, panadol, pentazocine, pimecrolimus, piroxicam, pregabalin, propoxyphene, quinine, remifentanil, rofecoxib, salsalate, sufentanil, sulfasalazine, sulindac, tramadol, valdecoxib, ziconotide
Antibiotic	133	acetylcysteine, acyclovir, adapalene, adefovir, amantadine, amikacin, amoxicillin, amphotericin, ampicillin, atovaquone, azithromycin, aztreonam, bacitracin, benzoyl peroxide, benzylpenicillin, caspofungin, cefaclor, cefadroxil, cefazolin, cefdinir, cefepime, cefixime, cefotaxime, cefotiam, cefpodoxime proxetil, cefprozil, ceftazidime, ceftriaxone, cefuroxime, cephalexin, chloramphenicol, chlorhexidine, chloroquine, ciclopirox, cilastatin, ciprofloxacin, clarithromycin, clindamycin, clotrimazole, colistin, cyclosporine, cytarabine, dactinomycin, dalacin, dapsone, daptomycin, daunorubicin, docosanol, doxycycline, econazole, entecavir, epirubicin, ertapenem, erythromycin, ethambutol, famciclovir, floxacillin, fluconazole, foscarnet, fosfluconazole, gamimune n, gammagard, ganciclovir, gatifloxacin, gemcitabine, gemifloxacin, gentamicin, gramicidin, hydroxychloroquine, idarubicin, imipenem, interferon alfacon-1, interferon gamma-1b, isoniazid, isotretinoin, itraconazole, ivermectin, ketoconazole, lansoprazole, levofloxacin, linezolid, mefloquine, meropenem, metronidazole, micafungin, miconazole, minocycline, moxifloxacin, mupirocin, neomycin, norfloxacin, nystatin, ofloxacin, oseltamivir, palivizumab, peg-interferon a-2a ro, penicillin, pentamidine isethionate, piperacillin, polymyxin b, posaconazole, povidone-iodine, primaquine, pyrazinamide, pyrimethamine, quinine, rapamycin, ribavirin, rifabutin, rifampin, rifaximin, roxithromycin, selenium sulfide, sodium polystyrene sulfonate, streptomycin, sulfadiazine, sulfamethoxazole, sulperazone, tazobactam, tazocilline, telithromycin, terbinafine, tetracycline, tigecycline, tioconazole, tobramycin, trimethoprim, valacyclovir, valganciclovir, vancomycin, virginiamycin, voriconazole, zanamivir
Anti-Depressant	32	amitriptyline, amoxapine, bupropion, citalopram, clomipramine, cyclobenzaprine, desipramine, dosulepin, doxepin, duloxetine, escitalopram, fluoxetine, fluvoxamine, imipramine, lithium carbonate, lofepramine, maprotiline, mianserin, mirtazapine, nefazodone, norepinephrine, nortriptyline, paroxetine, phenelzine, protriptyline, reboxetine, sertraline, sulpiride, tranylcypromine, trazodone, trimipramine, venlafaxine
Anti-inflammatory	58	abatacept, acetylsalicylate, acetylsalicylic acid, acitretin, adalimumab, allopurinol, alosetron, anakinra, azathioprine, balsalazide, betamethasone, celecoxib, chloroquine, clobetasol, colchicine, cortisone, deflazacort, desonide, dexamethasone, dipyrone, drotrecogin alfa, etanercept, etodolac, fludrocortisone, flunisolide, fluocinonide, flurbiprofen, glatiramer, hydrocortisone, hydroxychloroquine, ibuprofen, indomethacin, infliximab, interferon beta 1a, ketoprofen, ketorolac, leflunomide, mefenamic acid, meloxicam, mesalamine, methotrexate, methylprednisolone, mometasone, nabumetone, naproxen, nimesulide, oxaprozin, pimecrolimus, piroxicam, prednisolone, prednisone, probenecid, rofecoxib, salsalate, sulfasalazine, sulindac, triamcinolone, valdecoxib
Anti-psychotic	21	amisulpride, aripiprazole, atomoxetine, bromperidol, chlorpromazine, clozapine, fluphenazine, haloperidol, leponex, olanzapine, paliperidone, perphenazine, pipamperone, prochlorperazine, quetiapine, risperidone, sulpiride, thioridazine, thiothixene, trifluoperazine, ziprasidone
Anxiety	29	alprazolam, bromazepam, buspirone, carbamazepine, chlordiazepoxide, clobazam, clonazepam, clorazepate, diazepam, doxepin, doxylamine, duloxetine, estazolam, etomidate, flurazepam, fluvoxamine, gabapentin, hydroxyzine, lithium carbonate, lorazepam, meprobamate, midazolam, oxazepam, phenelzine, prazepam, temazepam, tranylcypromine, trazodone, triazolam
Cancer	99	aldesleukin, alemtuzumab, amifostine, anagrelide, anastrozole, arsenic, asparaginase, azacitidine, bevacizumab, bicalutamide, bleomycin, bortezomib, capecitabine, carboplatin, carmustine, cetuximab, cinacalcet, cisplatin, cladribine, clofarabine, cyclophosphamide, cytarabine, dacarbazine, dactinomycin, dasatinib, daunorubicin, decitabine, docetaxel, doxorubicin, durotep, dutasteride, epirubicin, erlotinib, estrone, estropipate, etoposide, exemestane, finasteride, fludarabine, fluorouracil, fulvestrant, gamimune n, gammagard, gefitinib, gemcitabine, gemtuzumab ozogamicin, goserelin, hydroxyurea, ibritumomab tiuxetan, idarubicin, ifosfamide, imatinib, imiquimod, interferon beta 1b, interferon gamma-1b, irinotecan, ixabepilone, lapatinib, lenalidomide, letrozole, leucovorin, leuprolide, megestrol, melphalan, mercaptopurine, mesna, methotrexate, mitomycin, mitoxantrone, oxaliplatin, paclitaxel, panitumumab, paricalcitol, pegaspargase, pegfilgrastim, peg-l-asparaginase, pemetrexed, pentostatin, rituximab, sargramostim, sorafenib, sunitinib, tamoxifen, tamsulosin, temozolomide, temsirolimus, terazosin, thalidomide, thioguanine, thiotepa, topotecan, toremifene, tositumomab, trastuzumab, tretinoin, vinblastine, vincristine, vinorelbine, vorinostat
Diabetes	21	acarbose, alpha-glucosidase, aspart, exenatide, gliclazide, glimepiride, glipizide, glucagon, glyburide, insulin, metformin, miglitol, nateglinide, octreotide, pioglitazone, pramlintide, protamine, repaglinide, rosiglitazone, sitagliptin, voglibose
Heart	110	abciximab, acebutolol, acenocoumarol, acetylsalicylate, acetylsalicylic acid, adenosine, alfuzosin, aliskiren, amiodarone, amlodipine, atenolol, atorvastatin, benazepril, bendroflumethiazide, betaxolol, bezafibrate, bisoprolol, bosentan, candesartan, captopril, carvedilol, cerivastatin, chlorthalidone, cholestyramine, cilostazol, clonidine, clopidogrel, colesevelam, colestipol, digitoxin, digoxin, diltiazem, dipyridamole, disopyramide, dobutamine, dofetilide, doxazosin, enalapril, eplerenone, epoprostenol, eprosartan, ezetimibe, felodipine, fenofibrate, flecainide, fluvastatin, fondaparinux, fosinopril, gemfibrozil, glimepiride, guanfacine, hydralazine, iloprost, ipratropium, irbesartan, isradipine, labetalol, latanoprost, lercanidipine, lidocaine, lisinopril, losartan, lovastatin, loxen, methyldopa, metolazone, metoprolol, mexiletine, midodrine, minoxidil, moexipril, moxonidine, nadolol, nebivolol, niacin, nicardipine, nicorandil, nifedipine, nisoldipine, nitric oxide, nitroglycerin, norepinephrine, olmesartan, pentoxifylline, perindopril, pindolol, pravastatin, prazosin, propafenone, propranolol, quinapril, ramipril, ranolazine, reteplase, rosuvastatin, simvastatin, sotalol, telmisartan, terazosin, ticlopidine, timolol, tirofiban, torsemide, trandolapril, treprostinil, trichlormethiazide, trimetazidine, ursodiol, valsartan, verapamil
Muscle Relaxant	45	acetazolamide, atracurium, baclofen, botox, carbamazepine, carisoprodol, cisatracurium, clidinium, clobazam, clonazepam, clorazepate, cyclobenzaprine, dantrolene, diazepam, divalproex, estazolam, ethosuximide, fosphenytoin, gabapentin, glycopyrrolate, lamotrigine, levetiracetam, metaxalone, methocarbamol, midazolam, orphenadrine, oxcarbazepine, pancuronium, phenobarbital, phenytoin, pregabalin, primidone, quinine, rocuronium, scopolamine, succinylcholine, temazepam, tetrazepam, tiagabine, tizanidine, topiramate, trihexyphenidyl, valproic acid, vecuronium, zonisamide
Obesity	7	dextroamphetamine, ephedrine, methamphetamine, orlistat, phentermine, phenylpropanolamine, sibutramine
Sleep	14	bromazepam, chlordiazepoxide, diphenhydramine, doxylamine, eszopiclone, etomidate, midazolam, oxazepam, quazepam, ramelteon, triazolam, zaleplon, zolpidem, zopiclone

Through cross-tabulation of the ISR numbers, each report supplied the patient's age, gender, and the nature of the reaction. For each drug, the patient ages and genders were summarized into mean patient age and percentage of males. The reactions were divided into “suicide attempt”, “suicide ideations”, “suicidal behavior”, and “other”. Because the meaning of “suicidal behavior” was not clear to us and such reports were small in number, they were excluded from regression models.

**Figure 1:**
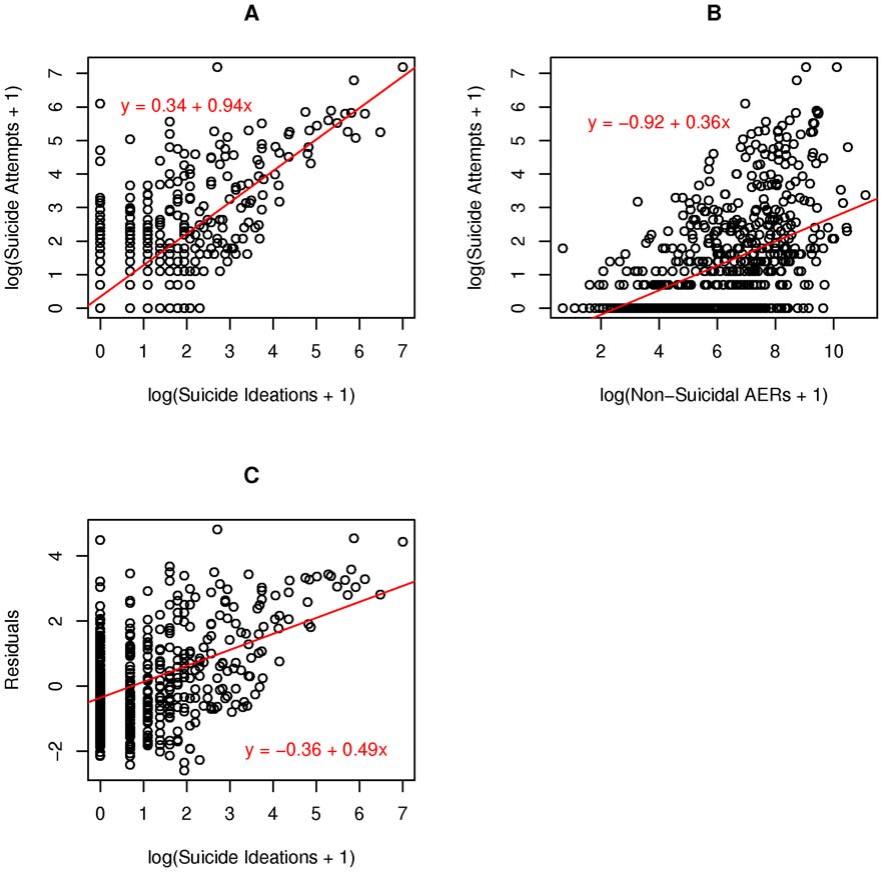
Log-log scatter plots.

As a first step, we made log-log scatter plots of suicidal attempts, ideations, and other AERs (Figure 1). Since many drugs had zero recorded suicide attempts (454 out of 832 drugs, or 55%), we plotted against the log of (suicide attempts +1), to avoid the situation where log(0) = −∞. As can be seen in panel A, when we ran a linear regression of the log counts, there was a clear positive association between suicide attempts and ideations. We also plotted suicide attempts against non-suicidal AERs (panel B), which showed that suicide attempts increased with the volume of usage. To isolate the association of ideation with attempts, we found the residuals of the linear regression from panel B and plotted them against attempts, which still showed a positive trend (p<.0001).

The association can be established more formally through the use of multiple regression. We fitted a variety of models, ranging from multiple linear regressions to generalized linear models, and all yielded consistent conclusions. Generalized linear models (GLMs) have an advantage over linear regression in that they accommodate the discrete nature of count data, as well as preventing the prediction of negative numbers. GLMs have been successfully applied to observational mental health studies involving count data [9]. A typical distribution used for count data GLMs is the Poisson distribution; however, in the Poisson distribution the mean of the count data equals its variance. Since we encountered severe overdispersion, in which the variance of the data was greater than the mean, we substituted the negative binomial distribution. The negative binomial substitution is a common technique applied to overdispersed data [10].

As covariates, we included drug class (e.g., antidepressant; diabetes drug, etc.), the mean age of patients, and the proportion of males who experienced AEs on a per-drug basis. Because age and gender may affect suicide risk, and could possibly create spurious associations between suicide ideation and attempts, they were included as covariates to adjust for these demographic factors. Because drug is the unit of analysis, it is average age and gender of those whose AE's reported by drug, rather than individual person age or gender that are modeled. To categorize the drugs into classes, we consulted drug information from the web-based Drug Information Portal at the U.S. National Library of Medicine. We used indicator variables for: antidepressant, anxiety, antipsychotic, heart, antibiotic, AIDS, cancer, allergies, analgesic, anti-inflammatory, diabetes, muscle relaxant, obesity, and sleep aid. For a given drug, membership in more than one class of drug was possible.

Among the reported reactions, we counted “completed suicide”, “suicide attempt”, “intentional overdose”, and “multiple drug overdose intentional” as suicide attempts. “Suicidal ideations” were treated as a separate predictor variable. There was another reaction, “suicidal behavior”, but since we could not determine whether this meant it was an attempt or an ideation, we did not count such reactions in the primary analysis. The 194 “suicidal behavior” reactions recorded accounted for only 0.01% of all reactions and were likely to be trivial in effect, so they were excluded from the primary analysis. However, in a sensitivity analysis, they were included as suicidal attempts. All other non-suicidal reactions were counted as “non-suicidal reactions”, a predictor variable.

Models were fitted in order of forward selection; starting with an intercept-only model that had no predictors, successive predictor variables were added one at a time. Each predictor's contribution to the proportion of variation in the outcome was estimated through Miaou's pseudo-R^2^ (see Appendix S1) [11]. In choosing which variable to add next, p-value alone was not sufficient, as several variables had p-values less than .0001. Thus, we chose the secondary criteria of its effect upon the pseudo-R^2^; variables that explained a greater proportion of variance were added first. In this way, non-suicidal AERs were chosen first, as they caused the largest increase in the pseudo-R^2^.

During the forward selection process, existing covariates whose p-values increased to greater than .05 were removed from the model. In the final stage, a Bonferroni correction was applied to remove covariates with p-values greater than .05/k, where k is the number of predictors in the model. This step eliminated variables whose p-values may have been an artifact of multiple testing.

## Results

### Descriptive Statistics

**Figure 2:**
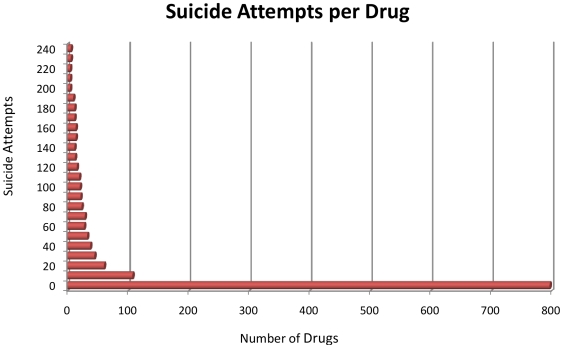
Distribution of suicide attempts per drug.

The distribution of reported suicide attempts per drug was highly positively skewed (Figure 2). That is, 690 out of 832 drugs (83%) had fewer than 10 attempts reported during this 4-year time period, while there was a long tail of drugs with 10 or more suicide attempts. The median number of attempts was 0 attempts per drug, while its SD was 84 (Table 3). On the high end, there were up to 1,323 suicide attempts out of 27,012 AERs (4.9%) for paroxetine. Suicide ideations followed a similar positively skewed distribution, with a median of 0 reported ideations per drug (SD = 57.5). On average, there were more attempts reported than ideations, although there were 135 drugs (16.2% of drugs) where the number of reported ideations was greater than attempts. Suicidal ideation would be expected to be considerably more common than suicidal acts, so there appears to be reporting bias here; the less serious outcomes appeared to be reported less often. Overall, suicide attempts and ideations were positively correlated (r = +.69, p<.0001); the strength of the correlation was stronger than that between suicide attempts and non-suicidal AERs (r = +.29, p<.0001). The positive association remained after adjusting for non-suicidal AERs and other variables in multiple regression models.

**Table 3:** Descriptive statistics by drug.

Variable	Mean	Min	1Q	Median	3Q	Max	SD
Number of suicide attempt reports per drug	17.2	0	0	0	5	1323	84
Number of suicide ideation reports per drug	9.5	0	0	0	2	1096	56
Number of non-suicidal reaction reports per drug	1661.1	1	35	235.5	1404	65683	4373
Percent of AE reports by males per drug	46.4%	0%	36%	48%	58%	100%	21%
Mean age of patients reporting AEs per drug	52.5	1.7	44.8	53.0	61.8	88.0	12.6

Table 4 provides descriptive statistics for other variables. The proportion of males who reported a reaction was as low as 0% for birth control drugs such as norethindrone, to as high as 99% for vardenafil, an erectile dysfunction drug. A total of 58 drugs (7.0% of all drugs) had a male proportion of 0% or 100%; some had gender-specific purposes, while others appeared to be artifacts of low sample sizes for the drugs.

**Table 4:** Model fit statistics.

Model	Dispersion Parameter	R_K_^2^	ΔR_K_^2^	p-value at entry
Intercept-only	8.79	0.000	–	<.0001
+ Other*	6.70	0.238	0.238	<.0001
+ Drug Class	4.68	0.468	0.230	<.0001
+ Gender	4.23	0.519	0.051	<.0001
+ Ideation	4.11	0.532	0.014	0.0096
+ Age	4.04	0.540	0.008	0.0217
+ Ideation × Other	3.88	0.558	0.018	<.0001
+ Drug Class × Ideation	3.84	0.564	0.005	0.0005
- Bonferroni Corrections	3.91	0.556	−0.008	–

* “Other” refers to non-suicidal AEs.

### Inferential Statistics

The changes in R^2^ as shown in Table 4 indicate that suicidal ideations were the single best predictor of suicide attempts, followed by drug class. Table 5 gives the details of the “full” model with all significant predictors included. For a negative binomial regression coefficient, the difference in the logs of expected counts of the response variable is expected to change by the respective regression coefficient for each unit increase in the predictor, given the other predictor variables in the model are held constant. Specifically, for every reported suicidal ideation per drug, attempts increased by a log count of .036, or a factor of 3.7% (p<.0001) with other factors held constant. Non-suicidal AERs also had a positive association with attempts (p<.0001), but had a much smaller association of 2.8% for every 100 AERs; a low p-value does not imply a large magnitude of association. Every reported ideation was associated with as many as 131 non-suicidal AERs, as inferred from the coefficients
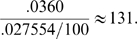



**Table 5:** Estimates of full model.

Variable	Raw Coefficient	Estimate*	SE	95% CI	p-value
Intercept	−3.699	0.025	1.042	(0.00, 0.19)	0.0004
Ideations	0.036	1.037	0.008	(1.02, 1.05)	<.0001
Other (per 10,000 AERs)	.0276	1.028	0.004	(1.02,1.04)	<.0001
Drug Class: Anxiety**	1.468	4.342	0.391	(2.02, 9.34)	0.0002
Drug Class: Antibiotic	−1.736	0.176	0.227	(0.11, 0.28)	<.0001
Drug Class: Cancer	−1.063	0.345	0.280	(0.20, 0.60)	0.0001
Drug Class: Analgesic	1.826	6.210	0.329	(3.26, 11.84)	<.0001
Drug Class: Anti-inflammatory	−1.614	0.199	0.350	(0.10, 0.40)	<.0001
% Male	0.0932	1.098	0.013	(1.07, 1.13)	<.0001
(% Male)^2^	−0.0009	0.999	0.000	(1.00, 1.00)	<.0001
Mean age	0.123	1.131	0.041	(1.04, 1.23)	0.0027
(Mean age)^2^	−0.0013	0.999	0.000	(1.00, 1.00)	0.0013
Ideations × Other	−0.0153	0.985	0.003	(0.98, 0.99)	<.0001
Ideations × Analgesic	−0.024	0.976	0.007	(0.96, 0.99)	0.0008

* This is the exponentiated raw coefficient; it is an estimate of the multiplicative factor for the outcome for every unit increase in the predictor.

** Drug classes were coded as indicator variables. It was possible for a given drug to belong to more than one drug class.

There did exist a negative interaction term between ideations and non-suicidal AEs; as the volume of non-suicidal AEs rose, each ideation was associated with a smaller increase in attempts.

Age and gender both had quadratic associations with suicide attempts with other factors held constant. The largest increase in suicide attempts were seen among drugs when the average age of patients was 47, and 52% of the reports were from men. This suggests that drugs used primarily by one gender, children, or old people were associated with fewer suicide attempts. Intuitively speaking, birth control pills, prostate drugs, acne medications, ADHD drugs, growth hormones, or Alzheimer medications are not typically associated with suicide attempts.

The sensitivity analysis in which “suicidal behavior” reactions were considered as suicidal attempts yielded essentially identical results to those of the primary analyses (data not shown).

## Discussion

In this paper, we used the strength of the enormous number of AE reports to estimate the extent to which drugs that have many suicidal ideations also tend to be drugs that have many suicide attempts or completions.

There are many limitations to the use of spontaneous AE reports, including questions about the quality and completeness of reporting, and the fact that denominator figures (i.e., total number of people exposed) were not freely available and therefore not utilized in our study. In this light, it is worth noting that an additional limitation is that drugs which have other common but non-suicide related adverse effects could make a ‘signal’ of suicide-related AE reports looks less noticeable, even when suicides are common in that drug. One source of denominator data that may help to overcome these limitations is IMS Health, and future research should consider merging such data with the type analyzed herein to evaluate the robustness of our findings [12]. Some drugs had a high proportion of suicide-related AEs, which was partially addressed through adjustments for drug class. The study did not attempt to identify individual drugs that presented a higher risk or volume of suicide-related AEs per se.

Analyses of AERs can serve as useful complements to RCTs. RCTs, though more rigorous, generally do not have sufficient sample sizes to provide estimates of key effects and associations with rare events. That being said, it is important to note that our unit of analysis was drug and not person. Thus, our results have no direct bearing on the extent to which suicidal ideations among individual persons are predictive of suicidal attempts or completions among individual persons. The complex interaction between suicidal attempts and ideations at an individual level is a topic of ongoing research [13]. By adjusting for the total volume of AERs and demographic variables, it is possible to determine whether there remains a statistical association between suicidal ideation and attempts.

Our analysis shows that after adjusting for non-suicidal AERs, drug class, and demographic variables on a per drug basis, each reported suicidal ideation was associated with a 0.035 increase in the log count of suicide attempts, or a 3.7% increase. These results support the wisdom of the recent withdrawal of rimonabant from the market and the discontinuation of CB1 antagonist development for obesity research by multiple pharmaceutical companies including Merck, Pfizer, Solvay, and others. This further suggests, but does not prove, that drug-induced suicidal ideations in RCTs may indeed be indicators of a drug that is likely to increase suicidal attempts and/or completions for some individuals and not be merely self-limiting reversible epiphenomena.

## Supporting Information

Appendix S1 Miaou's Pseudo R^2^.
(0.02 MB DOC)
