# Supplementary material for: Drugs Associated with More Suicidal Ideations Are also Associated with More Suicide Attempts
Source: PLoS One. 2009 Oct 2;4(10):e7312. doi: 10.1371/journal.pone.0007312 (PMC2749439; doi:10.1371/journal.pone.0007312)
Supplement: Appendix S1 — Miaou's Pseudo R2. (0.02 MB DOC) [file pone.0007312.s001.doc]

## Appendix 1. Miaou’s Pseudo R^2^.

Miaou proposed a relatively simple formula for expressing the explanatory power of variables via an R^2^–like metric for generalized linear models.^10^ Generalized linear models differ from least-square models in that R^2^ cannot be estimated directly, since the fitting is done through an iterative algorithm to maximize likelihood. In the formula below, K refers to the value of the overdispersion parameter for a given negative binomial model; K_max_ is the overdispersion parameter for an intercept-only model.

$$R_{K}^{2}=1 - \frac{K}{K_{max}}$$
